# Supplementary material for: Fructose-Induced Metabolic Dysfunction Is Dependent on the Baseline Diet, the Length of the Dietary Exposure, and Sex of the Mice
Source: Nutrients. 2024 Dec 31;17(1):124. doi: 10.3390/nu17010124 (PMC11722689; doi:10.3390/nu17010124)
Supplement: Supplementary file 1 [file nutrients-17-00124-s001.zip › Supplemental Figures _11-19-24.pdf]

Supplement Fig 1

A.

	9F 5020 Diet BCD	T2018 Diet LXD	D12450K Diet LFD
Calorie Density (kcal/g)	3.46	3.1	3.85
Net Metab Energy	3.18	2.78	3.71
% Protein Calories	23	24	20
% Fat Calories	22	18	10
% Carb Calories	55	58	70

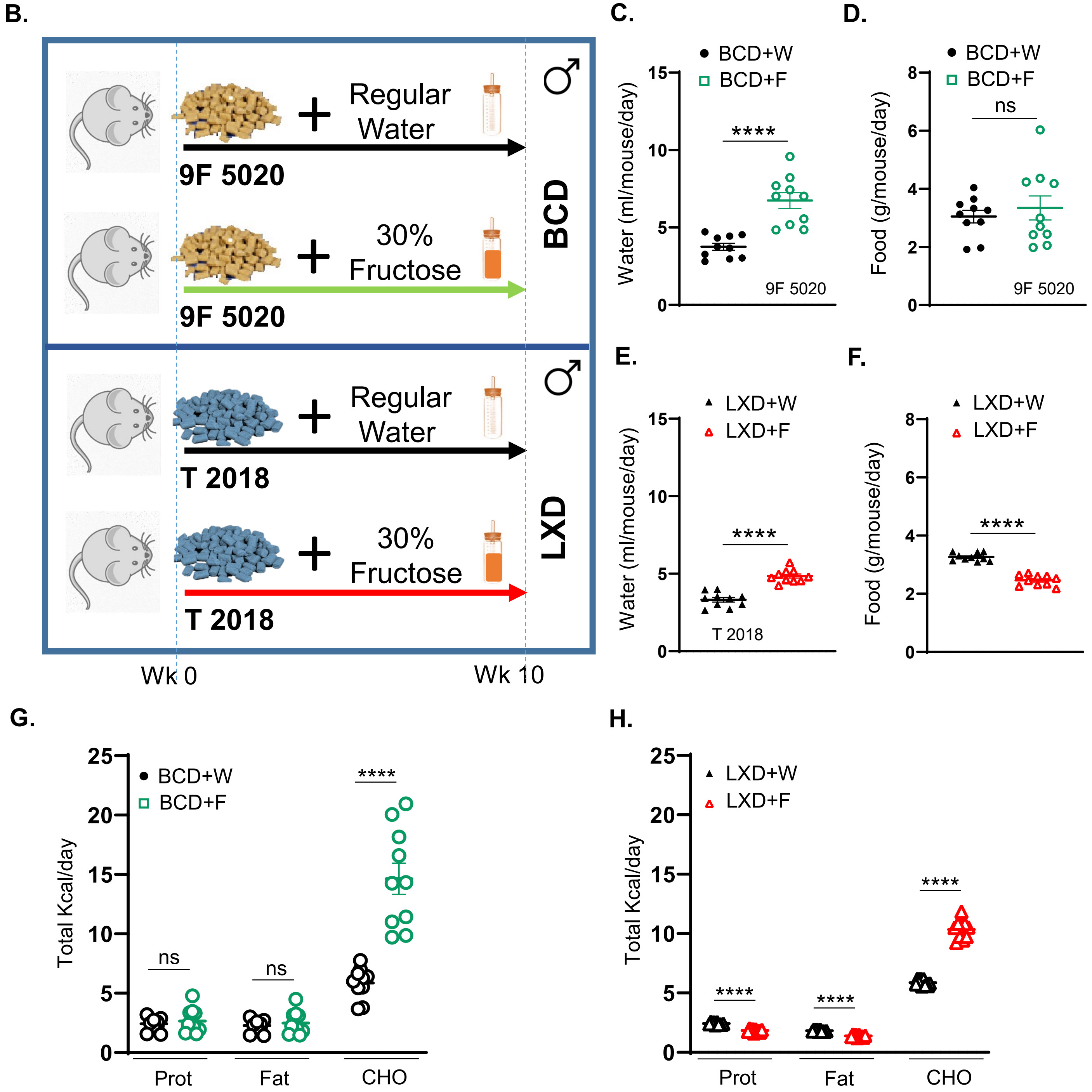

Supplemental Figure 1
A) Diet composition of Boston Chow Diet (BCD), Lexington Chow Diet (LXD), and Low Fat Diet (LFD). **B)** Experimental design for male mice on BCD and LXD. **C)** Water consumption, and **D)** Food intake of mice fed BCD for 10 weeks. **E)** Water consumption, and **F)** Food intake of mice fed LXD for 10 weeks. Caloric intake from protein (prot), fat and charbohydrate (CHO) in mice on **G)** BCD and **H)** LXD. n=8 mice per group. Each data point represents an average of two cages per one week. There were four mice per cage and each group contained two cages of mice. Intake data was divided by 4 mice and 7 days to get g/mouse/day. Statistical comparisons were conducted using Student’s t-test for pairwise comparisons between the control and fructose groups. Statistical significance is indicated by asterisks (*) when comparing fructose-supplemented groups to their respective controls: *$p < 0.05$; **$p < 0.01$; ***$p < 0.001$; ****$p < 0.0001$. Data are presented as mean ± SEM.

Supplement Fig 2

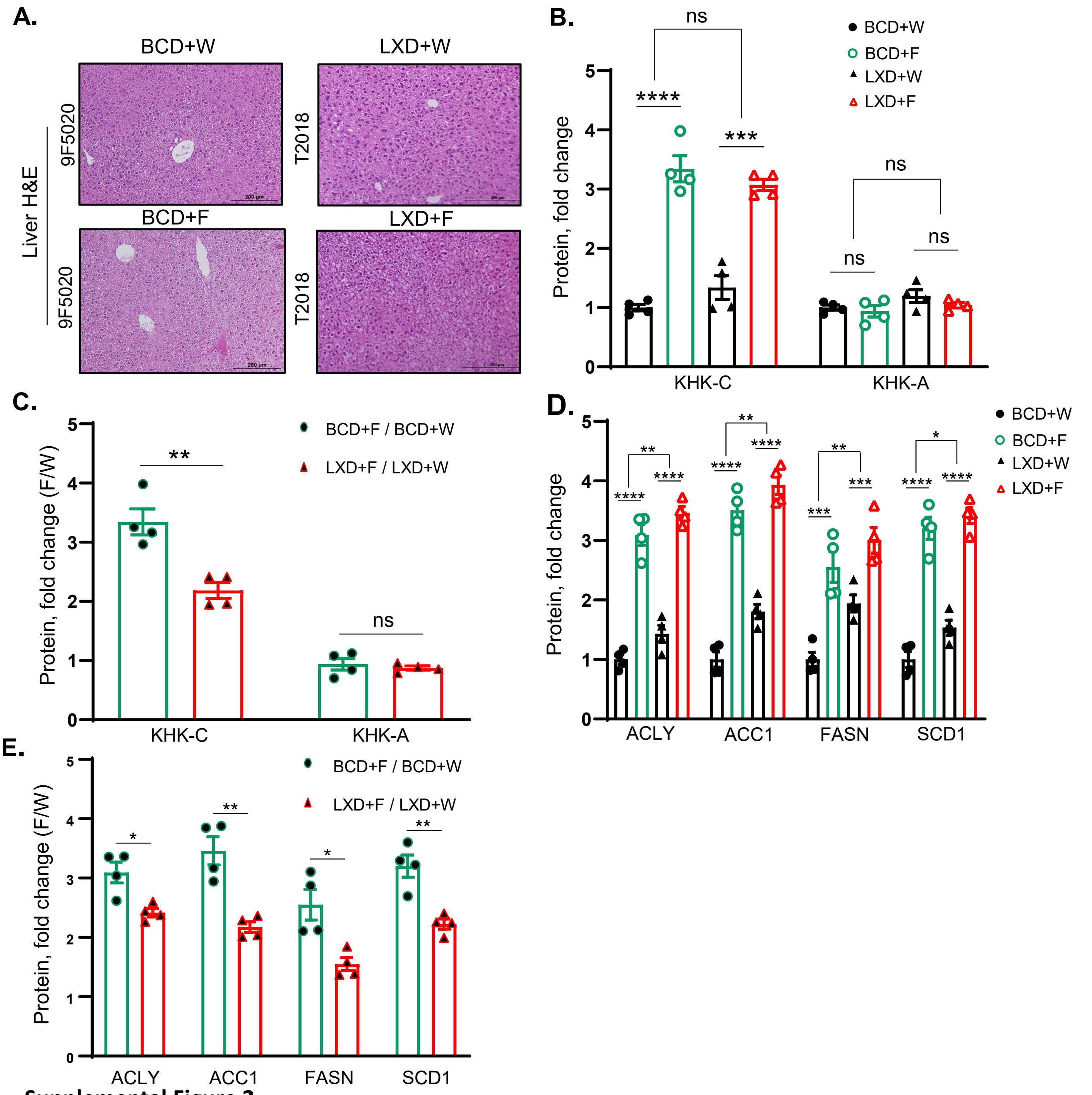

Supplemental Figure 2

A) Hematoxylin and Eosin (H&E) stained histology in male mice on BCD and LXD supplemented with fructose or regular water. **B)** Image J quantification of protein levels of enzymes that regulate the first step of fructose metabolism from Western blot shown in Fig. 2H **C)** KHK-C and KHK-A fold-change induction with fructose baselined to the water group on BCD and LXD. **D)** Image J quantification of protein levels of enzymes involved in fatty acid synthesis in the liver from Western blot shown in Fig. 2K. **E)** Hepatic fatty acid synthesis enzymes fold-change induction with fructose baselined to the water group on BCD and LXD. Statistical analysis was performed using two-way ANOVA followed by Tukey's post hoc test for comparisons across multiple groups. Statistical significance, denoted by asterisks (*), represents comparisons of fructose-supplemented groups to their respective controls, as follows: *$p < 0.05$; **$p < 0.01$; ***$p < 0.001$; ****$p < 0.0001$. All data are expressed as mean ± SEM.

Supplement Fig 3

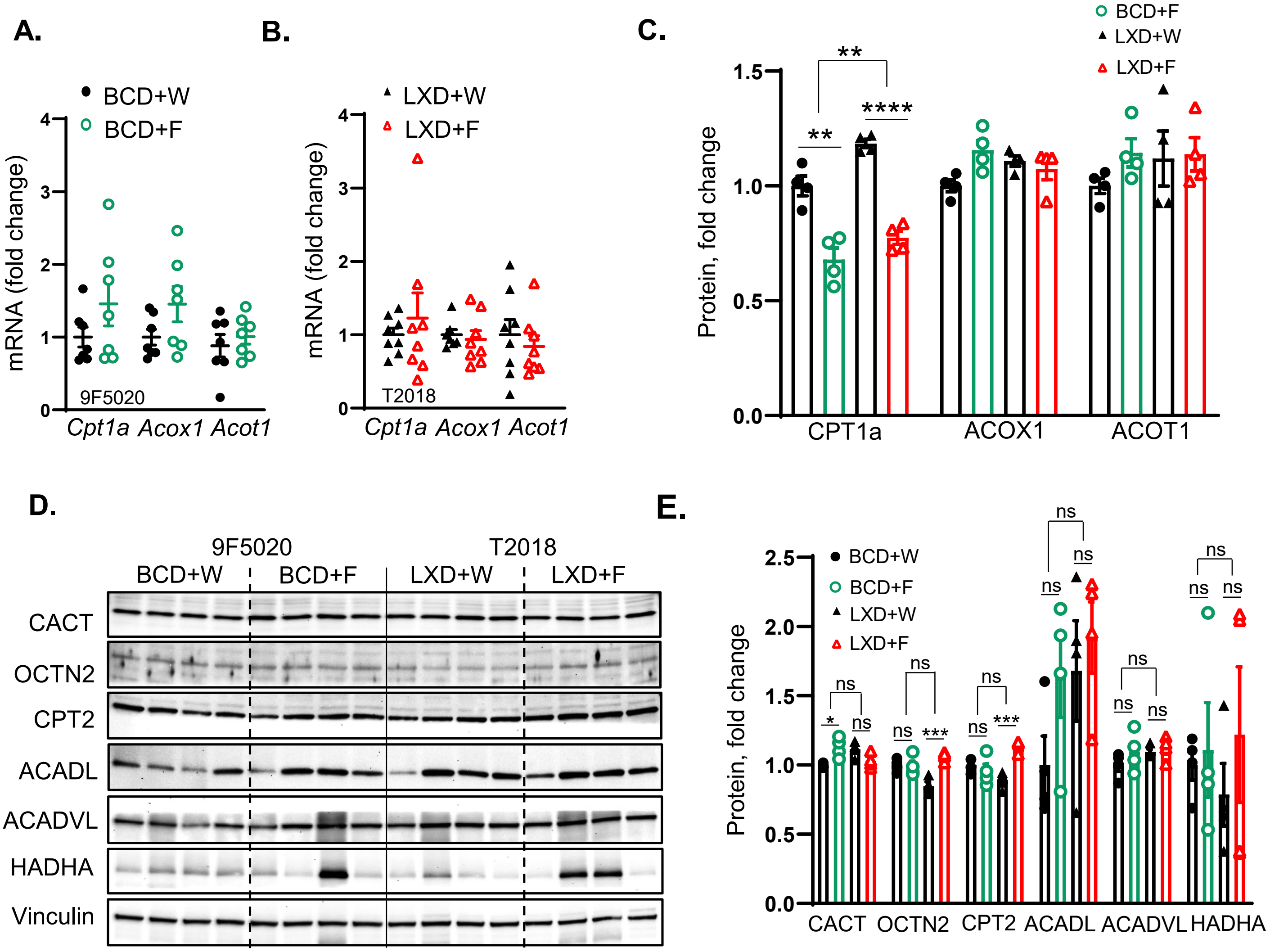

Supplemental Figure 3

A) mRNA expression of enzymes involved in hepatic fatty acid oxidation in the livers of mice on BCD, **B)** and LXD provided fructose or regular water. **C)** Image J quantification of protein levels of enzymes involved in hepatic fatty acid oxidation on BCD and LXD from Western blot shown in Fig. 2L . **D)** Protein levels of additional enzymes involved in hepatic fatty acid oxidation in BCD and LXD. **E)** Image J quantification of protein levels of enzymes involved in hepatic fatty acid oxidation from Western blot shown in Sup Fig. 3D. Statistical analysis was performed using two-way ANOVA followed by Tukey's post hoc test for comparisons across multiple groups. Statistical significance, denoted by asterisks (*), represents comparisons of fructose-supplemented groups to their respective controls, as follows: *p < 0.05; **p < 0.01; ***p < 0.001; ****p < 0.0001. All data are expressed as mean ± SEM.

Supplement Fig 4

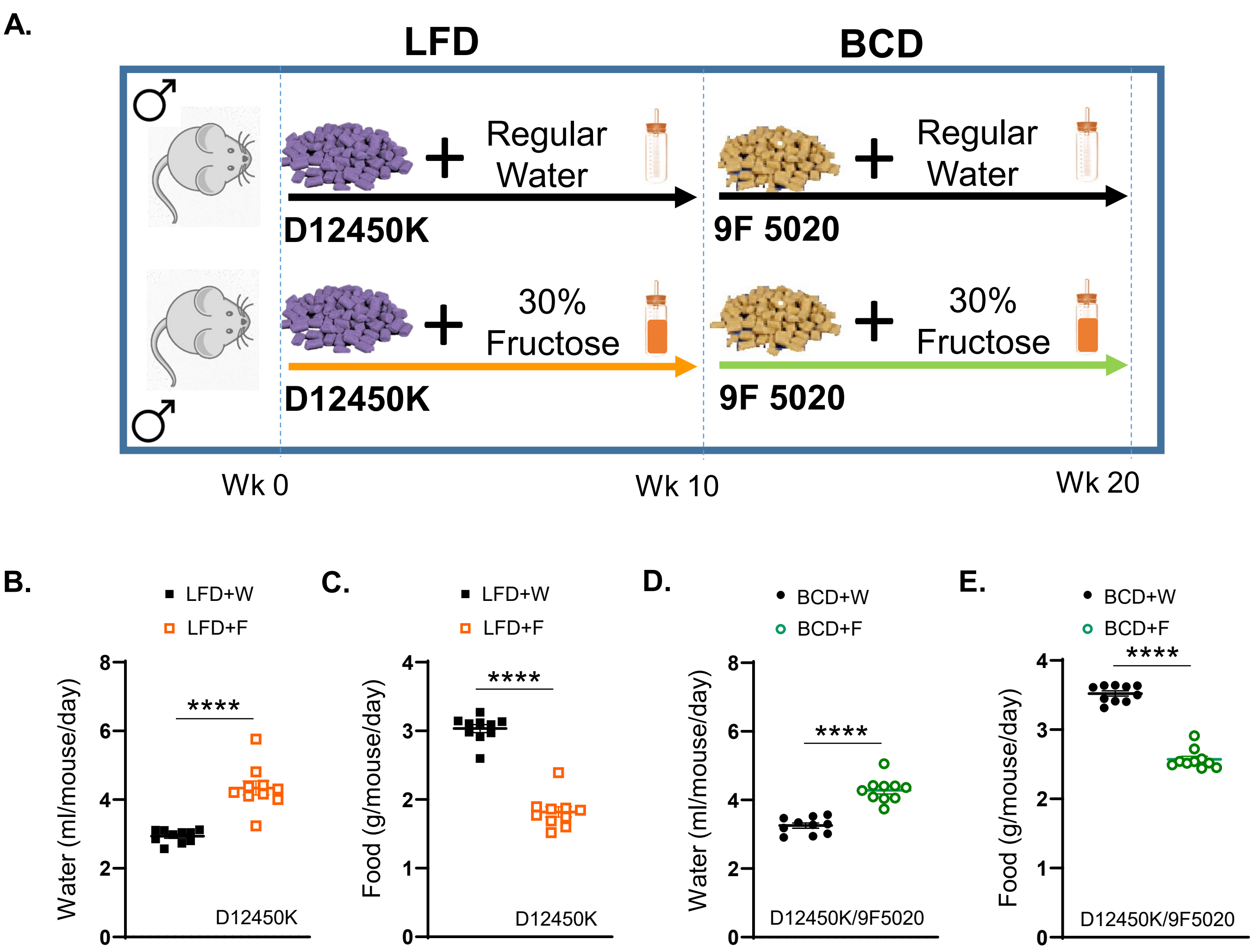

Supplemental Figure 4

A) Experimental design for male mice on Low Fat Diet (LFD) for 10 weeks and then crossed over to Boston Chow Diet (BCD) for another 10 weeks and provided with fructose or regular water. **B)** Water consumption, and **C)** Food intake of LFD-fed mice for 10 weeks on the diet. **D)** Water consumption and **E)** Food intake of BCD-fed mice for an additional 10 weeks on the diet. n=8 mice per group. There were four mice per cage and each group contained two cages of mice. Water and food intake data was divided by 4 mice and 7 days to get g/mouse/day. Statistical comparisons were conducted using Student's t-test for pairwise comparisons between the control and fructose groups. Statistical significance is indicated by asterisks (*) when comparing fructose-supplemented groups to their respective controls: *$p < 0.05$; **$p < 0.01$; ***$p < 0.001$; ****$p < 0.0001$. Data are presented as mean ± SEM.

Supplement Fig 5

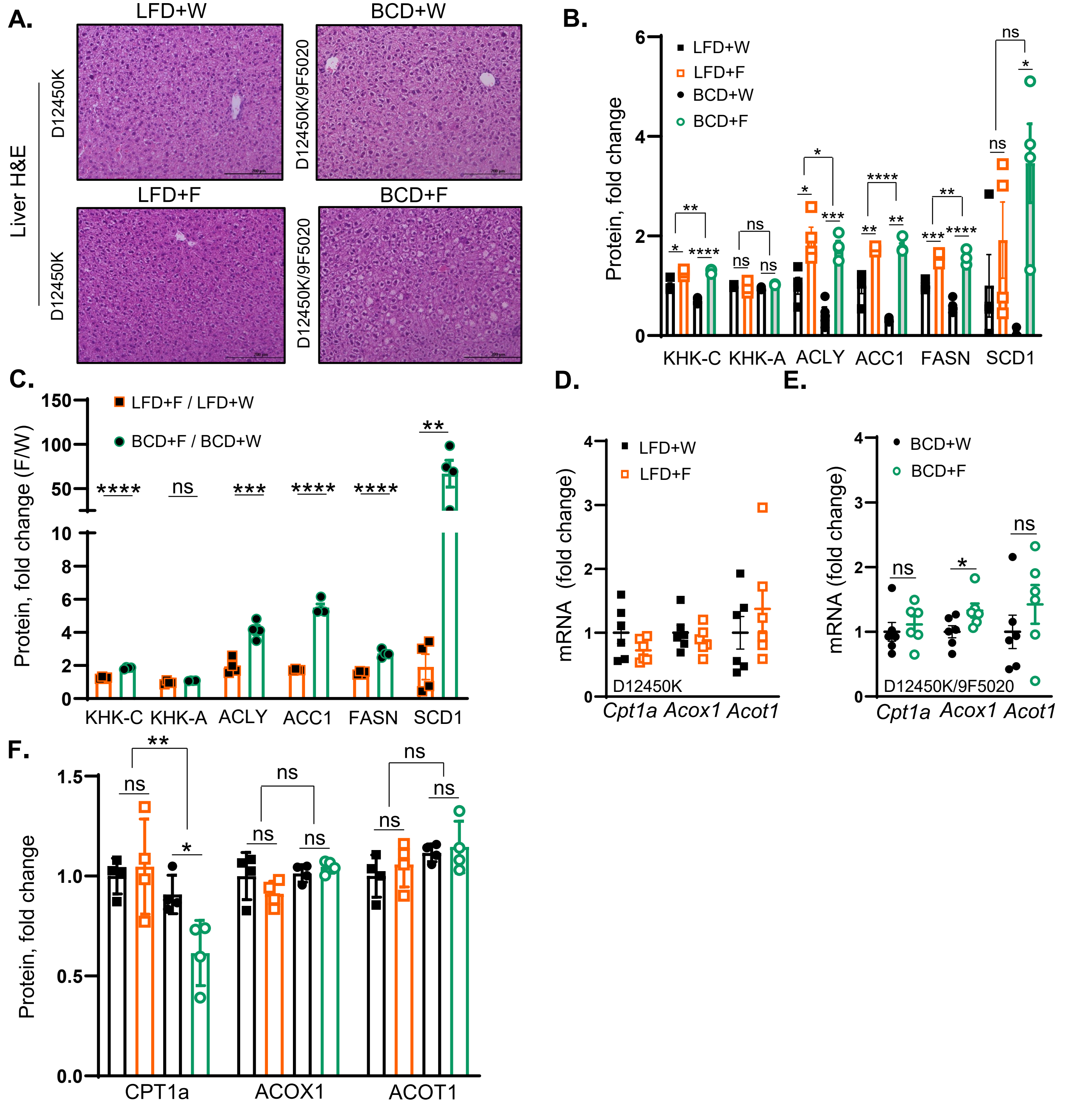

Supplemental Figure 5

A) Hematoxylin and Eosin (H&E) histology of male mice on LFD and BCD, provided with regular or fructose water. **B)** Image J quantification of protein levels of enzymes involved in the first step of fructose metabolism and in hepatic fatty acid synthesis from Western blot shown in Figs. 4H and 4K. **C)** KHK and hepatic fatty acid synthesis enzymes fold-change induction with fructose baselined to the water group. **D)** mRNA expression of enzymes involved in hepatic fatty acid oxidation on LFD, **E)** and BCD. **F)** Image J quantification of protein levels of enzymes involved in hepatic fatty acid oxidation from Western blot shown in Fig. 4L. Statistical analysis was performed using two-way ANOVA followed by Tukey's post hoc test for comparisons across multiple groups. Statistical significance, denoted by asterisks (*), represents comparisons of fructose-supplemented groups to their respective controls, as follows: *$p < 0.05$; **$p < 0.01$; ***$p < 0.001$; ****$p < 0.0001$. All data are expressed as mean ± SEM.

Supplement Fig 6

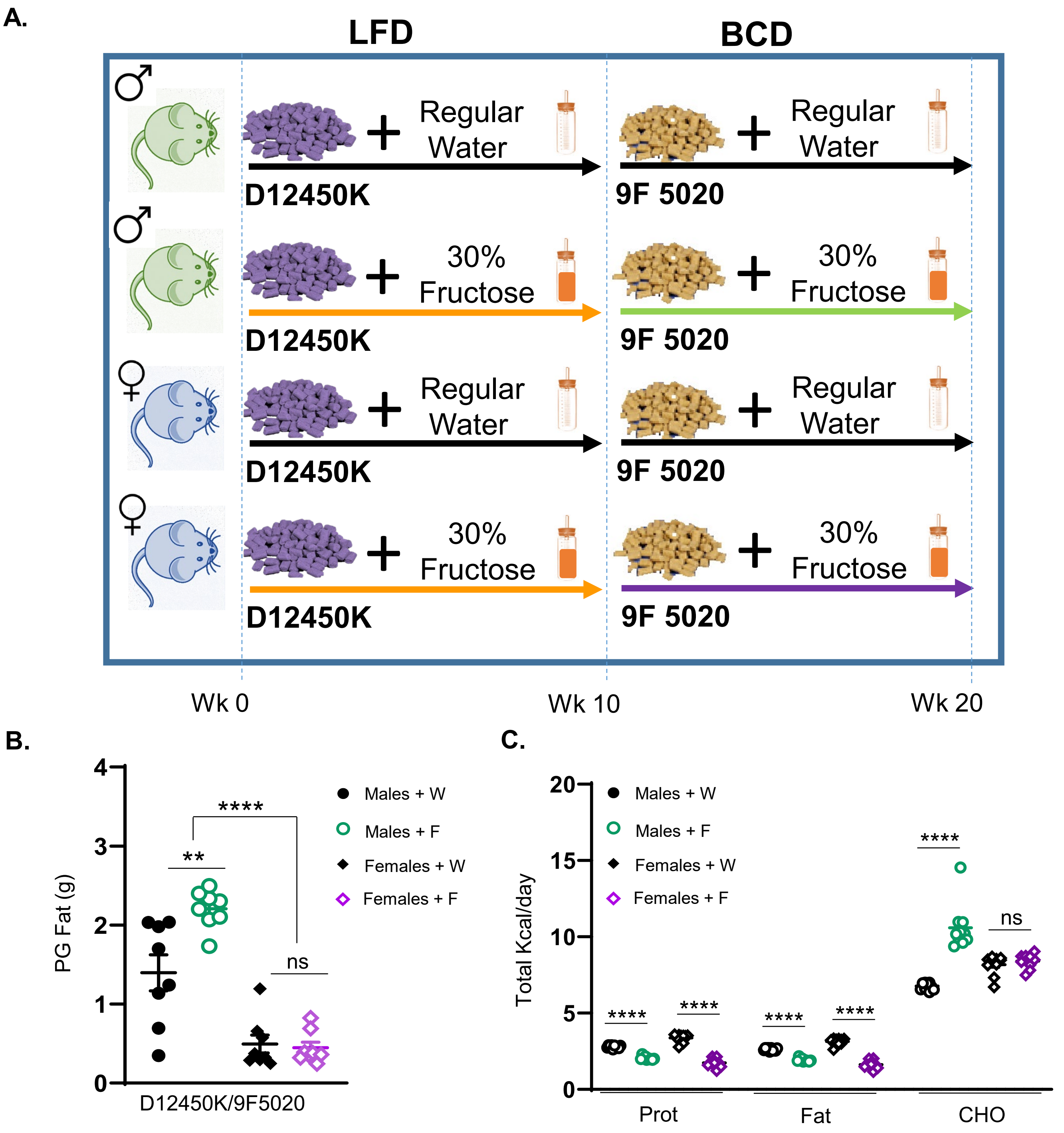

Supplemental Figure 6
A) Experimental design for male and female mice on LFD for 10 weeks and then crossed over to BCD for an additional 10 weeks. The mice were provided regular or fructose water. **B)** Mass of perigonadal (PG) adipose tissue in male and female mice supplemented with fructose (F) or regular water (W). **C)** Caloric intake from protein (prot), fat and carbohydrate (CHO). Statistical analysis was performed using two-way ANOVA followed by Tukey's post hoc test for comparisons across multiple groups. Statistical significance, denoted by asterisks (*), represents comparisons of fructose-supplemented groups to their respective controls, as follows: *$p < 0.05$; **$p < 0.01$; ***$p < 0.001$; ****$p < 0.0001$. All data are expressed as mean ± SEM.

Supplement Fig 7

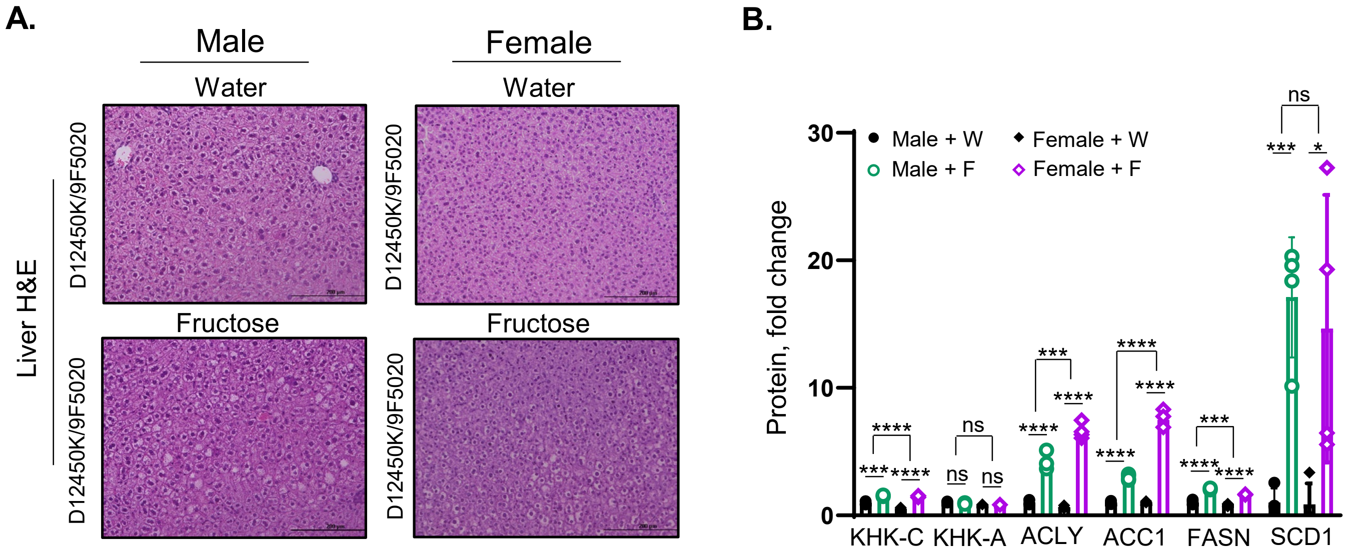

Supplemental Figure 7

A) Hematoxylin and Eosin (H&E) stained histology of male and female mice after 20 weeks on the diets and provided with regular or fructose water. **B)** Image J quantification of protein levels of enzymes involved in fructolysis and hepatic fatty acid synthesis of male and female mice, from Western blot shown in Fig. 6F and 6G. Statistical analysis was performed using two-way ANOVA followed by Tukey's post hoc test for comparisons across multiple groups. Statistical significance, denoted by asterisks (*), represents comparisons of fructose-supplemented groups to their respective controls, as follows: *$p < 0.05$; **$p < 0.01$; ***$p < 0.001$; ****$p < 0.0001$. All data are expressed as mean ± SEM.
