# Supplementary material for: Fructose-Induced Metabolic Dysfunction Is Dependent on the Baseline Diet, the Length of the Dietary Exposure, and Sex of the Mice
Source: Nutrients. 2024 Dec 31;17(1):124. doi: 10.3390/nu17010124 (PMC11722689; doi:10.3390/nu17010124)
Supplement: Supplementary file 1 [file nutrients-17-00124-s001.zip › Supplemental tables-11-19-24.pdf]

Supplementary tables

Table S1. Mouse primers used for real-time quantitative PCR.

Gene	Sequence (5' – 3')	Forward/Reverse
Acly	CAGCCAAGGCAATTTCAGAGC	Forward
	CTCGACGTTTGATTAACTGGTCT	Reverse
Acc1	ACAGTGGAGCTAGAATTGGAC	Forward
	ACTTCCCGACCAAGGACTTTG	Reverse
Fasn	GGAGGTGGTGATAGCCGGTAT	Forward
	TGGGTAATCCATAGAGCCCAG	Reverse
Scd1	CAGCCGAGCCTTGTAAGTTC	Forward
	GCTCTACACCTGCCTCTTCG	Reverse
Khk-c	AACTCCTGCACTGTCCTTTCCTT	Forward
	CCACCAGGAAGTCGGCAA	Reverse
Cpt1a	AGTGGCCTCACAGACTCCAG	Forward
	GCCCATGTTGTACAGCTTCC	Reverse
Khk-a	TTGCCGATTTTGTCCTGGAT	Forward
	CCTCGGTCTGAAGGACCACAT	Reverse
18S	GTAACCCGTTGAACCCCATT	Forward
	CCATCCAATCGGTAGTAGCG	Reverse
Tbp	TGACTGCAGCAAATCGCTTGG	Forward
	ACCCTTCACCAATGACTCCTATG	Reverse
ACOX1	ACTCGCAGCCAGCGTTATG	Forward
	AGGGTCAGCGATGCCAAAC	Reverse
ACOT1	AGAGGAAGAGTTGGGCAGAG	Forward

	TTCGTCCCAGCAGCAGCG	Reverse

Table S2. Antibodies used for Western Blot.

Name	Citation	Supplier	Cat no.
Rabbit polyclonal anti-ACLY	RRID: AB_2223744	Cell Signaling Technology	4332
Rabbit monoclonal anti-ACC	RRID: AB_2219397	Cell Signaling Technology	3676
Rabbit monoclonal anti-FASN	RRID: AB_2100796	Cell Signaling Technology	3180
Rabbit polyclonal anti-SCD1	RRID: AB_823634	Cell Signaling Technology	2438
Rabbit monoclonal anti-KHK-A		Signal way Antibody	21708
Rabbit monoclonal anti-KHK-C		Signal way Antibody	21709
Mouse monoclonal anti-CPT1a	RRID: AB_11141632	Abcam	ab128568
Mouse monoclonal anti-Vinculin	RRID: AB_2272814	Novus Biologicals	NB600-1293
Rabbit polyclonal anti-OCTN2	RRID: AB_2191406	Proteintech	16331-1-AP
Rabbit polyclonal anti-CACT	RRID: AB_10642001	Proteintech	19363-1-AP

Rabbit polyclonal anti-CPT2	RRID: AB_2084849	Abcam	ab71435
Mouse monoclonal anti-ACADVL	RRID: AB_10609094	Santa-Cruz	sc-271225
Rabbit polyclonal anti-ACADL	RRID: AB_1859818	Abcam	ab82853
Mouse monoclonal anti-Actin	RRID: AB_476744	Sigma	A5441
Rabbit monoclonal anti-pAkt	RRID: AB_2315049	Cell Signaling Technology	4060
Rabbit monoclonal anti-total Akt	RRID: AB_915783	Cell Signaling Technology	4691
Rabbit monoclonal anti-pERK	RRID: AB_2315112	Cell Signaling Technology	4370
Rabbit monoclonal anti-total ERK	RRID: AB_330744	Cell Signaling Technology	9102
Mouse monoclonal anti-HADHA	RRID: AB_ 10862577	Abcam	ab110302
Mouse monoclonal anti-ACOX1	RRID: AB_ 3075447	Santa-Cruz	sc-517306
Mouse monoclonal anti-ACOT1	RRID: AB_10918465	Santa-Cruz	sc-373917
